# Conditional 1-year outcomes after emergent conversion to open heart surgery during transcatheter aortic valve implantation: a propensity-matched landmark analysis

**DOI:** 10.3389/fcvm.2025.1660381

**Published:** 2025-09-18

**Authors:** Giuseppe Nasso, Walter Vignaroli, Gaetano Contegiacomo, Alfredo Marchese, Ernesto Greco, Khalil Fattouch, Raffaele Bonifazi, Flavio Fiore, Giacomo Schinco, Antongiulio Valenzano, Carlo Solimando, Vito Margari, Fabrizio Resta, Tommaso Loizzo, Dritan Hila, Domenico Paparella, Giuseppe Speziale

**Affiliations:** ^1^ Department of Cardiac Surgery, Anthea Hospital and Santa Maria Hospital GVM Care & Research, Bari, Italy; ^2^ Department of Cardiac Surgery, San Carlo di Nancy Hospital GVM Care & Research, Rome, Italy; ^3^ Department of Health and Life Sciences, European University of Rome, Rome, Italy; ^4^ Department of Cardiac Surgery, Maria Eleonora Hospital GVM Care & Research, Palermo, Italy; ^5^ Department of Cardiac Surgery, University of Foggia, Foggia, Italy

**Keywords:** TAVI, surgical bailout, surgical standby, aortic valve replacement, TAVR

## Abstract

**Background:**

Emergent conversion to open heart surgery (E-OHS) during transcatheter aortic valve implantation (TAVI) is rare (0.5%–2%) but carries high perioperative mortality. Long-term outcomes in survivors beyond 30 days are not well defined.

**Objectives:**

To assess 1-year conditional outcomes in patients who survived ≥30 days post-TAVI, comparing E-OHS survivors with propensity-matched uncomplicated TAVI recipients.

**Methods:**

Between January 2020 and August 2023, 825 consecutive TAVI procedures were performed at three Italian centers; 11 patients (1.3%) required E-OHS for catastrophic intraprocedural complications. A 30-day landmark analysis excluded early deaths (E-OHS: *n* = 3; controls: *n* = 25). Propensity matching (1:10) was performed on nine variables, yielding 8 E-OHS survivors and 80 well-matched controls.

**Primary endpoint:**

All-cause mortality from day 31 to 1 year.

**Secondary endpoints:**

Composite of death, moderate-or-greater paravalvular regurgitation, or valve reintervention; heart failure rehospitalization; permanent pacemaker; stroke/transient ischemic attack (TIA); and acute kidney injury (AKI).

**Results:**

Baseline characteristics were comparable. The mean age was 77 ± 5 years; EuroSCORE II was 6.8 ± 2.1%. One-year conditional mortality was 0% in E-OHS survivors vs. 2.9% in controls (*p* = 0.64). The composite endpoint occurred in 12.5% vs. 13.6% (*p* = 0.88). Other outcomes were similar: heart failure rehospitalization (12.5% vs. 11.2%), pacemaker implantation (12.5% vs. 9.6%), stroke/TIA (0% vs. 1.2%), and AKI (0% vs. 7.2%). No structural valve deterioration or thrombosis was observed.

**Conclusions:**

E-OHS survivors who overcome the initial high-risk phase achieve 1-year outcomes comparable to standard TAVI patients. These findings support immediate surgical backup within TAVI programs and provide reassurance for high-risk patient counseling.

## 1 Introduction

Severe aortic stenosis (AS) affects an ever-growing elderly population and is associated with dismal prognosis if untreated (1). Transcatheter aortic valve implantation (TAVI) has revolutionized its management, demonstrating non-inferiority or superiority to surgical aortic valve replacement (SAVR) across extreme-, high-, intermediate-, and low-risk cohorts (2–4). Global adoption has now surpassed 5,00,000 implants, with nearly 70% of aortic valve replacements in the United States performed percutaneously in 2024. Nevertheless, TAVI is not exempt from life-threatening complications. Annular rupture, coronary obstruction, ventricular perforation, aortic dissection, and device embolization may precipitate hemodynamic collapse, demanding *emergent conversion to open heart surgery* (E-OHS) (5–7).

### 1.1 Contemporary landscape of E-OHS

The reported prevalence of E-OHS has remained stable (0.5%–2%) despite advancements in less-invasive techniques, including the miniaturization of delivery systems and the use of less-traumatic guidewires. This paradox reflects two opposing trends: reduced hardware-related injury thanks to low-profile catheters, and expansion of TAVI into anatomically complex, low-risk, and often bicuspid valves that were once exclusive to surgery. The widest meta-analysis of 37 studies (*n* ≈ 40,000) confirmed an E-OHS incidence rate of 1.1% yet highlighted substantial between-center variability, suggesting that team experience and infrastructure, more than patient mix, dictate conversion rates (8).

### 1.2 Early vs. late hazard paradigm

Early registry data reported E-OHS perioperative mortality rate surpassing 50% (8). Technological advances, systematic computed tomography planning, and hybrid operating suites have reduced both frequency and lethality, yet published evidence still concentrates on 30-day outcomes (9–11). Whether E-OHS confers an enduring penalty beyond this early hazard remains unknown and constitutes a critical knowledge gap for patient counseling and programmed design. Cardiac surgery literature distinguishes an *early* hazard phase—procedure-specific and modifiable—from a *late* hazard governed by comorbidity and valve durability. Applying this paradigm to TAVI may illuminate the true prognostic impact of E-OHS.

### 1.3 Knowledge gaps and study rationale

Two conceptual hurdles obscure mid-term appraisal. First, E-OHS patients exhibit a distinctly bimodal risk curve: a steep early hazard and an indeterminate late phase. Pooling early and late events may distort true conditional prognosis. Second, E-OHS patients inherently differ from standard TAVI recipients with respect to anatomy, procedural complexity, and hemodynamic insult, introducing confounding. Propensity-score methods mitigate such imbalances but are rarely coupled with landmark analysis. Furthermore, contemporary innovations—such as cusp-overlap fluoroscopy, commissural alignment strategies, and per-procedural hemodynamic modeling—may influence both the incidence and the outcome of E-OHS, yet they remain understudied.

### 1.4 Objectives

Herein, we performed a 30-day landmark and 1:10 propensity-matched comparison of conditional 1-year outcomes following E-OHS vs. uncomplicated TAVI in a contemporary multi-institutional cohort. We hypothesized that once the perioperative window (30 days) is cleared, E-OHS survivors experience survival and morbidity comparable to matched controls. In addition, we sought to identify the clinical predictors of conditional mortality and to contextualize findings within the evolving TAVI ecosystem, including cost-effectiveness, decentralization debates, and guideline updates.

## 2 Methods

### 2.1 Study design and oversight

This retrospective, observational study utilized prospectively maintained databases from three Italian Heart-Valve Centers inside the same network “GVM Care & Research”: Anthea Hospital, Bari; Santa Maria Hospital, Bari; and San Carlo di Nancy, Rome. All centers provide on-site 24/7 cardiac surgery treatment. All clinical data were retrospectively extrapolated by our general and cumulative registry database (containing clinical information of all patients admitted to the hospitals) and then retrospectively analyzed. The study conforms to the ethical principles of Good Clinical Practice, the Helsinki Declaration, and complies with the current regulations. All patients gave written informed consent for inclusion, collection/use of data or samples, and/or publication according to the actual guidelines. The IRB number for the research protocol is TB001-2.

### 2.2 Patient selection and definitions

Between January 2020 and August 2023, 825 consecutive TAVI procedures were performed. The inclusion criteria were as follows: transfemoral or alternative access TAVI and complete 1-year follow-up. E-OHS was defined as unplanned emergent sternotomy or thoracotomy with or without the need for cardiopulmonary bypass (CPB) during the index procedure for hemodynamic crash. Device success and complications were adjudicated per Valve Academic Research Consortium-3 (VARC-3) criteria (12).

### 2.3 Procedural protocol

The procedure was always performed in a catheter laboratory. A complete standby cardiac surgery rescue team equipped to emergency management for E-OHS was always present during the procedure.

Details of the TAVI procedures are described elsewhere (13).

An anesthesiologist was always present during TAVI, and during E-OHS, the procedures were all converted to general anesthesia with orotracheal intubation. The E-OHS is always performed with an immediate sternotomy and direct cardiac resuscitation in patients without any history of cardiac surgery. In those with previous cardiac surgery, an extra corporeal membrane oxigenation (ECMO) is positioned peripherally and then resternotomy is performed. A maximum of 20 min was necessary to guarantee circulation support in both procedures.

We used 4 types of aortic prostheses: in 448 patients (54.3%), “Corevalve Evolut R” (Medtronic, Minneapolis, MN, USA), in 281 patients (34.1%), a “Portico” (Abbott Vascular, Santa Clara, CA, USA), in 94 patients (11.4%), a “Corevalve” (Medtronic, Minneapolis, MN, USA), and in 2 patients (0.2%), a “Myval” (Meril Life Sciences Pvt. Ltd., Vapi, Gujarat, India).

The average prosthesis size was 27.5 mm, with the most common dimensions being 26 mm (120 cases, 21%), 27 mm (71 cases, 12.5%), and 29 mm (159 cases, 28%). Prior to valve implantation, 42 patients (5.1%) underwent PCI to treat significant concomitant coronary artery disease. In 12 patients (1.4%), the transfemoral approach was not feasible; consequently, 6 trans-subclavian TAVI procedures (0.7%) were performed with surgical artery exposure, and 6 trans-axillary TAVI procedures (0.7%) were performed with a percutaneous approach.

Thirteen patients (1.6%) developed cardiogenic shock: 2 were treated with pharmacological support, and 11 underwent E-OHS.

Two patients with postprocedural complications were excluded from the emergency group: one developed cardiac tamponade 24 h later because of subtle ventricular damage, while the other experienced severe mitral regurgitation related to systolic anterior movement one week after the procedure.

### 2.4 Follow-up and data collection

Before discharge and at 1, 6, and 12 months, patients underwent clinical evaluation, New York Heart Association (NYHA) classification, and transthoracic echocardiography (TTE). The rate of data completeness exceeded 97%. Mortality status was cross-checked through the national registry.

### 2.5 Landmark and propensity-score matching

Thirty-day mortality amounted to 3/11 E-OHS (27%) and 25/814 controls (3.1%), as described elsewhere (13). These patients were excluded, defining a *conditional cohort* of 8 E-OHS and 789 controls alive at day 30. Propensity scores estimating the likelihood of E-OHS were calculated via multivariable logistic regression encompassing age, sex, body mass index, EuroSCORE II, left-ventricular ejection fraction (LVEF), chronic kidney disease (estimated glomerular filtration rate (eGFR) < 60mL/min/1.73 m^2^), NYHA class III–IV, access route, and valve type. One E-OHS survivor was matched with up to ten controls using nearest-neighbor, caliper 0.2 SD of the logit. Covariate balance was judged adequate when the standardized mean difference (SMD) <0.1 for all variables.

### 2.6 Study endpoints

Primary endpoint: all-cause mortality from landmark (day 31) to 1 year. Secondary endpoints: composite of death, mitral regurgitation (MR) ≥moderate (grade 2) or valve reintervention, heart failure rehospitalization; new permanent pacemaker (PPM), stroke or transient ischemic attack (TIA), and acute kidney injury (AKI) ≥ KDIGO stage 1.

### 2.7 Statistical analysis

Continuous variables were presented as mean ± SD or median (interquartile range (IQR)) and compared with paired Student's t or Wilcoxon signed-rank tests. Categorical variables were counts (percentage) analyzed using McNemar or Bowker symmetry tests. Kaplan–Meier estimators described time-to-event distributions; differences were assessed with log-rank tests. Hazard ratios (HRs) with 95% confidence intervals (CIs) were derived from univariable Cox models. Proportional hazards assumption was confirmed via Schoenfeld residuals. Missing data (<3%) were imputed using expectation maximization algorithms. Analyses were executed in R 4.3.2 (MatchIt, survival, survminer); *p* < 0.05 denoted significance.

## 3 Results

### 3.1 Procedural characteristics and early outcomes

Of the 825 procedures, 814 (98.7%) were completed without surgical escalation and 11 (1.3%) required E-OHS. All E-OHS patients were treated for life-threatening complications: ventricular free wall perforation in seven patients (64%), acute left main coronary occlusion after valve release in two patients (18%), valve embolization with retrograde aortic dissection in one (9%), and mitro-aortic continuity disruption in one (9%). The median time from complication recognition to skin incision was 12 min (IQR: 9–16) and the total skin-to-CPB initiation was 14 min (IQR: 10–18). The mean cardiopulmonary bypass time in the E-OHS patients was 62 ± 18 min, and an aortic cross-clamp was required in three patients (27%). Thirty-day outcomes are detailed in Table 1: early mortality 3/11 (27%) in E-OHS vs. 25/814 (3.1%) in controls (*p* < 0.001) and prolonged ventilation >24 h 55% vs. 6% (*p* < 0.001).

**Table 1:** Early 30-day outcomes (Entire Cohort).

Outcome	E-OHS (*n* = 11)	Non-E-OHS (*n* = 814)	*p*-value
Mortality 30 days	27% (3)	3.1% (25)	<0.001
Major bleeding	82% (9)	10% (79)	<0.001
Prolonged ventilation >24 h	55% (6)	6% (49)	<0.001
Stroke/TIA	0%	1.2% (10)	0.64
AKI ≥ Stage 1	45% (5)	8% (65)	<0.001

### 3.2 Conditional cohort construction and baseline balance

After exclusion of early deaths (30 days), the landmark cohort consisted of 8 E-OHS survivors and 789 non-E-OHS survivors. Propensity score distribution demonstrated good overlap. The final 1:10 match yielded 8 E-OHS and 80 controls with negligible residual imbalance: age 78.6 ± 5.95 vs. 78.4 ± 4.7 years; EuroSCORE II 6.9 ± 2.0% vs. 6.7% ± 2.2% (SMD 0.04). Echocardiographic parameters such as baseline LVEF (50% ± 7% vs. 52% ± 6%) and aortic valve area (0.68 ± 0.15 cm^2^ both) were likewise comparable (Table 2).

**Table 2:** Baseline characteristics after propensity matching (*n* = 77).

Variable	E-OHS (*n* = 8)	Matched controls (*n* = 80)	SMD
Age (years)	78.6 ± 5.95	78.4 ± 4.7	0.04
Female	43%	46%	0.06
EuroSCORE II (%)	6.9 ± 2.0	6.7 ± 2.2	0.04
LVEF (%)	50 ± 7	52 ± 6	0.03
CKD (eGFR < 60)	29%	30%	0.02
NYHA III–IV	57%	54%	0.06

### 3.3 Primary outcome: conditional 1-year survival

**Figure 1:**
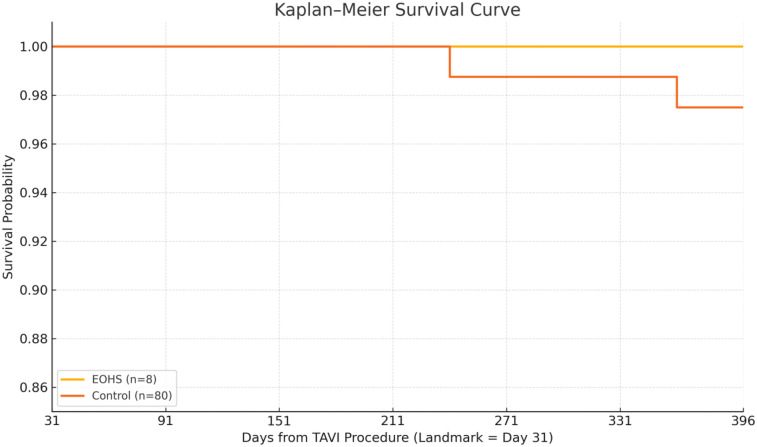
Survival probability: the Kaplan–Meier curve displays a near-complete overlap; the absolute risk difference at 12 months was −2.9% (95% CI: −9.3% to 3.5%). Log-rank *p* = 0.64; HR for death E-OHS vs. controls 0.33 (95% CI: 0.02–5.64).

During 339 patient months of conditional follow-up, no E-OHS survivor died, whereas two control patients succumbed to their disease conditions—one died of septic shock 7 months’ post-TAVI, and the other died of progressive heart failure at 11 months. The Kaplan–Meier curve (Figure 1) displays near-complete overlap; the absolute risk difference at 12 months was −2.9% (95% CI: −9.3% to 3.5%). Log-rank *p* = 0.64; HR for death E-OHS vs. controls 0.33 (95% CI: 0.02–5.64).

### 3.4 Secondary clinical endpoints

*Heart failure rehospitalization* occurred in one E-OHS patient (12.5%, day 176 post procedure, responsive to diuretics) and in nine matched controls (11.2%, *p* = 0.94). *Permanent pacemaker implantation* spanning between 30 days and 1 year was required in one E-OHS patient (new complete AV block at 3 months) vs. eight controls (9.6%, *p* = 0.81). *Stroke/TIA* was rare: none in E-OHS patients and one TIA in controls. In E-OHS patients, there was no *acute kidney injury* ≥ *stage 1*, but this condition developed in 6 controls (7.2%; *p* = 0.34).

The composite endpoint of death, MR ≥ grade 2 or valve reintervention occurred in 1 E-OHS patient (12.5%)—a moderate paravalvular leak managed conservatively at 9 months—and in 11 controls (13.6%) (*p* = 0.88) (Table 3).

**Table 3:** Conditional 30 days → 1-year clinical outcomes (matched cohort).

Endpoint	E-OHS (*n* = 8)	Controls (*n* = 80)	*p*-value
All-cause death	0%	2.5% (2)	0.64
Composite death/MR ≥ 2/reintervention	12.5% (1)	13.6% (11)	0.88
Heart failure (HF) rehospitalization	12.5% (1)	11.2% (9)	0.94
Permanent pacemaker	12.5% (1)	9.6% (8)	0.81
Stroke/TIA	0%	1.2% (1)	1.00
AKI ≥ stage 1	0%	7.2% (6)	0.34

### 3.5 Echocardiographic evolution

Mean-indexed aortic valve gradient at 12 months was 12 ± 4 mmHg in both groups. Paravalvular leak at follow-up was graded none/trace in 71%, mild in 14%, and moderate in 14% (1 case) among E-OHS vs. none/trace 68%, mild 29%, and moderate 3%, among controls (*p* = 0.29). No cases of structural valve deterioration or valve thrombosis were detected.

### 3.6 Predictors of conditional mortality in the entire landmark cohort (*n* = 796)

A univariable Cox analysis identified EuroSCORE II > 8% (HR: 2.7; 95% CI: 1.1–6.7; *p* = 0.04) and LVEF < 40% (HR: 3.4; 95% CI: 1.3–8.5; *p* = 0.02) as significant predictors. Neither E-OHS status (HR: 0.9; *p* = 0.89) nor access route, valve type, baseline NYHA class, or chronic kidney disease (CKD) reached statistical significance. Interaction testing showed no modification of the E-OHS effect across EuroSCORE strata (*p*-interaction = 0.71).

## 4 Discussion

Our landmark propensity-matched analysis provides novel insights into the mid-term prognosis of patients undergoing emergent surgical bailout during TAVI. Three principal findings merit emphasis.

1. **Successful bailout abolishes excess conditional risk.** Despite a perioperative mortality rivaling major cardiac surgery, E-OHS survivors displayed 1-year survival identical to matched uncomplicated TAVI recipients. This pattern mirrors the concept of “hazard phase separation” described in SAVR cohorts, where early operative deaths are distinct from late attrition governed by comorbidity rather than procedure. Clinically, it means that devastated hemodynamics and extended CPB do not impose sustained biological damage if the patient crosses the 30-day threshold. Notably, durability signals such as the absence of structural valve deterioration or thrombosis among E-OHS survivors support the notion that the prosthetic environment remains stable even after bailout surgery.
2. **Institutional preparedness is pivotal.** All E-OHS events occurred with a complete rescue team on standby, yielding a skin-to-bypass time of approximately 14 min—inside the 20 min threshold associated with survival in experimental models (14). Emerging data show that centers without an on-site rescue team rely on delayed transfer, a scenario incompatible with the time-critical nature of annular rupture or coronary occlusion (15). Our findings reinforce ESC 2021 recommendations requiring immediate surgical availability for structural heart interventions (15). Cost-effectiveness analyses suggest that preventing a single E-OHS death offsets the incremental expense of maintaining surgical standby in high-volume centers (16), thereby strengthening the health economics argument for integrated programs.
3. **Propensity-matched landmark methodology clarifies outcomes.** Prior registry publications aggregated early deaths with late survivors, inevitably portraying E-OHS as a marker of poor long-term prognosis (17). By isolating the conditional cohort, we separated procedure-related lethality from patient-related natural history, revealing equivalence—a message crucial for informed consent and the mental wellbeing of rescued patients.

### 4.1 Comparison with the literature and emerging technologies

Pineda et al. analyzed the cases of 47,546 TAVI patients in the STS/ACC TVT registry: The E-OHS incidence rate was 1.2% with 50% 30-day mortality but lacked conditional follow-up (8). Li et al. reported 35% 30-day mortality rate and 45% 2-year survival rate among 20 E-OHS patient cases without matching (9).

Verolino et al. (19) analyzed 17,473 patients, demonstrating an incidence of major intraprocedural complications during TAVI procedures managed with E-OHS of approximately 1.3%, and they found that the urgent need for E-OHS was burdened by relevant procedural deaths (over 20% in the first 48 h and 50% within 12 months).

Our study, leveraging contemporary devices, hybrid workflows, and rigorous matching, provides the first evidence of conditional equivalence. Moreover, modern procedural refinements—cusp-overlap fluoroscopy to minimize perforation risk, commissural alignment to facilitate coronary reaccess, and AI-driven CT segmentation to predict annular rupture—may further reduce E-OHS incidence or improve salvage. Incorporating such variables into future risk-prediction models could refine preprocedural planning and patient selection.

### 4.2 Implications for decentralization and volume–outcome relationship

Advocates for expanding TAVI to centers without a complete rescue team argue that technological maturity renders bailout obsolete. Our data contradict this narrative: although rare, E-OHS remains a deadly event unless managed immediately, and survivors enjoy normalized prognosis only when salvaged promptly. Volume–outcome studies corroborate that high-volume centers deliver lower E-OHS rates and superior bailout success (18). Policymakers should consider mandating minimum procedural volumes and on-site surgical capability before accrediting new TAVI programs.

### 4.3 Future directions

Prospective registry harmonization with core-laboratory imaging and biorepository linkage could elucidate mechanisms underlying late valve performance in E-OHS patients. Machine learning algorithms integrating hemodynamic waveform analysis and intraoperative imaging may provide early warning of catastrophic complications, allowing preemptive mitigation. Randomized trials of cerebral embolic protection and rapid ECMO deployment in high-risk anatomies are warranted. Finally, patient-reported outcome measures (PROMs) focused on anxiety and quality-of-life post bailout would capture the often overlooked psychosocial dimension.

## 5 Limitations

A retrospective design with a modest E-OHS sample size limited multivariable modeling in this study. Matching could not account for unmeasured confounders (e.g., frailty phenotype). Echocardiography lacked core-laboratory adjudication; however, interobserver variability was moderated by shared training. Finally, the results reflected high-volume centers and they may not be generalized to low-volume hospitals lacking surgical infrastructure.

## 6 Conclusions

Thirty-day survivors of E-OHS during TAVI demonstrate 1-year survival, valve durability, and clinical outcomes indistinguishable from propensity-matched uncomplicated TAVI controls. Efficient surgical bailout therefore neutralizes the 1-year impact of catastrophic intraprocedural complications. Ensuring immediate availability of cardiac surgery and perfusion support remains essential to modern TAVI programs.

## Data Availability

The raw data supporting the conclusions of this article will be made available by the authors without undue reservation.

## References

[1] Nkomo VT Gardin JM Skelton TN Gottdiener JS Scott CG Enriquez-Sarano M. Burden of valvular heart diseases: a population-based study. Lancet. (2006) 368:1005–11. 10.1016/S0140-6736(06)69208-8 16980116

[2] Mack MJ Leon MB Thourani VH Makkar R Kodali SK Russo M Transcatheter aortic-valve replacement with a balloon-expandable valve in low-risk patients, N Engl J Med. (2019) 380:1695–705. 10.1056/NEJMOA1814052/SUPPL_FILE/NEJMOA1814052_DATA-SHARING.PDF 30883058

[3] Popma JJ Deeb GM Yakubov SJ Mumtaz M Gada H O’Hair D Transcatheter aortic-valve replacement with a self-expanding valve in low-risk patients, N Engl J Med. (2019) 380:1706–15. 10.1056/NEJMOA1816885/SUPPL_FILE/NEJMOA1816885_DATA-SHARING.PDF 30883053

[4] Reardon MJ Van Mieghem NM Popma JJ Kleiman NS Søndergaard L Mumtaz M Surgical or transcatheter aortic-valve replacement in intermediate-risk patients. N Engl J Med. (2017) 376:1321–31. 10.1056/NEJMOA1700456/SUPPL_FILE/NEJMOA1700456_DISCLOSURES.PDF 28304219

[5] Eggebrecht H Vaquerizo B Moris C Bossone E Lämmer J Czerny M Incidence and outcomes of emergent cardiac surgery during transfemoral transcatheter aortic valve implantation (TAVI): insights from the European Registry on Emergent Cardiac Surgery during TAVI (EuRECS-TAVI). Eur Heart J. (2018) 39:676–84. 10.1093/EURHEARTJ/EHX713 29253177

[6] Marin-Cuartas M de Waha S Naumann S Deo SV Kang J Noack T Incidence and outcomes of emergency intraprocedural surgical conversion during transcatheter aortic valve implantation: insights from a large tertiary care centre. Eur J Cardio-Thoracic Surg. (2023) 63:ezad142. 10.1093/ejcts/ezad142 37027228

[7] Généreux P Piazza N Alu MC Nazif T Hahn RT Pibarot P Valve academic research consortium 3: updated endpoint definitions for aortic valve clinical research. J Am Coll Cardiol. (2021) 77:2717–46. 10.1016/j.jacc.2021.02.038 33888385

[8] Pineda AM Harrison JK Kleiman NS Rihal CS Kodali SK Kirtane AJ Incidence and outcomes of surgical bailout during TAVR: insights from the STS/ACC TVT registry. JACC Cardiovasc Interv. (2019) 12:1751–64. 10.1016/j.jcin.2019.04.026 31537276

[9] Li F Wang X Wang Y Li X Zhao S Wu Y Short-and long-term outcome after emergent cardiac surgery during transcatheter aortic valve implantation. Ann Thorac Cardiovasc Surg. (2021) 27:112–8. 10.5761/atcs.oa.20-00123 33455973 PMC8058541

[10] Otto CM Nishimura RA Bonow RO Carabello BA Erwin JP Gentile F 2020 ACC/AHA guideline for the management of patients with valvular heart disease: executive summary: a report of the American College of Cardiology/American Heart Association joint committee on clinical practice guidelines. Circulation (2021) 143:e35–71. 10.1161/CIR.0000000000000932 33332149

[11] Vahanian A Beyersdorf F Praz F Milojevic M Baldus S Bauersachs J 2021 ESC/EACTS guidelines for the management of valvular heart disease: developed by the task force for the management of valvular heart disease of the European Society of Cardiology (ESC) and the European Association for Cardio-Thoracic Surgery (EACTS). Rev Esp Cardiol. (2022) 75:524. 10.1016/j.rec.2022.05.006 35636831

[12] Leipsic J Gurvitch R Labounty TM Min JK Wood D Johnson M Multidetector computed tomography in transcatheter aortic valve implantation. JACC Cardiovasc Imaging. (2011) 4:416–29. 10.1016/J.JCMG.2011.01.014 21492818

[13] Nasso G Vignaroli W Contegiacomo G Marchese A Fattouch K D’Alessandro P Emergent conversion to open heart surgery during transcatheter aortic valve implantation: the presence of a rescue team improves outcomes. J Clin Med. (2023) 12:7705. 10.3390/JCM12247705 38137774 PMC10743555

[14] Fernandes P Cleland A Bainbridge D Jones PM Chu MWA Kiaii B. Development of our TAVI protocol for emergency initiation of cardiopulmonary bypass. Perfus (United Kingdom). (2015) 30:34–9. 10.1177/0267659114547754 25143415

[15] Eggebrecht H Bestehorn M Haude M Schmermund A Bestehorn K Voigtländer T Outcomes of transfemoral transcatheter aortic valve implantation at hospitals with and without on-site cardiac surgery department: insights from the prospective German aortic valve replacement quality assurance registry (AQUA) in 17 919 patients. Eur Heart J. (2016) 37:2240–8. 10.1093/EURHEARTJ/EHW190 27190093

[16] Kim WK Tamburino C Möllmann H Montorfano M Ellert-Gregersen J Rudolph TK Clinical outcomes of the ACURATE neo2 transcatheter heart valve: a prospective, multicentre, observational, post-market surveillance study. EuroIntervention. (2023) 19:83–92. 10.4244/EIJ-D-22-00914 PMC10173758 36440588

[17] Arsalan M Kim WK Van Linden A Liebetrau C Pollock BD Filardo G Predictors and outcome of conversion to cardiac surgery during transcatheter aortic valve implantation. Eur J Cardio-Thoracic Surg. (2018) 54:267–72. 10.1093/EJCTS/EZY034 29506158

[18] Liao YB Deng XX Meng Y Zhao ZG Xiong TY Meng XJ Predictors and outcome of acute kidney injury after transcatheter aortic valve implantation: a systematic review and meta-analysis. EuroIntervention. (2017) 12:2067–74. 10.4244/EIJ-D-15-00254 27890858

[19] Verolino G Di Mauro M Calderone D Lorusso R. Major intraprocedural complications during transcatheter aortic valve implantation requiring emergent cardiac surgery: an updated systematic review. Am J Cardiol. (2025) 247:21–8. 10.1016/j.amjcard.2025.03.031 40174696

